# New *Olig1* null mice confirm a non-essential role for Olig1 in oligodendrocyte development

**DOI:** 10.1186/1471-2202-15-12

**Published:** 2014-01-14

**Authors:** Joana Paes de Faria, Nicoletta Kessaris, Paul Andrew, William D Richardson, Huiliang Li

**Affiliations:** 1 Wolfson Institute for Biomedical Research and Research Department of Cell and Developmental Biology, University College London, Gower Street, London WC1E 6BT, UK; 2 Present address: Instituto de Biologia Molecular e Celular, Rua do Campo Alegre, 823, 4150-180, Porto, Portugal

**Keywords:** Oligodendrocyte, Olig1, Olig2, Myelin, Knockout mice, Spinal cord, Forebrain

## Abstract

**Background:**

*Olig1* and *Olig2*, encoding closely related basic helix-loop-helix transcription factors, were originally identified in screens for glial-specific genes. *Olig1* and *Olig2* are both expressed in restricted parts of the neuroepithelium of the embryonic spinal cord and telencephalon and subsequently in oligodendrocyte lineage cells throughout life. In the spinal cord, Olig2 plays a crucial role in the development of oligodendrocytes and motor neurons, and both cell types are lost from *Olig2* null mutant mice. The role of Olig1 has been more cryptic. It was initially reported that *Olig1* null mice (with a *Cre-Pgk-Neo* cassette at the *Olig1* locus) have a mild developmental phenotype characterized by a slight delay in oligodendrocyte differentiation. However, a subsequent study of the same line following removal of *Pgk-Neo* (leaving *Olig1-Cre*) found severe disruption of oligodendrocyte production, myelination failure and early postnatal lethality. A plausible explanation was proposed, that the highly expressed *Pgk-Neo* cassette in the original line might have up-regulated the neighbouring *Olig2* gene, compensating for loss of Olig1. However, this was not tested, so the importance of Olig1 for oligodendrocyte development has remained unclear.

**Results:**

We generated two independent lines of *Olig1* null mice. Both lines had a mild phenotype featuring slightly delayed oligodendrocyte differentiation and maturation but no long-term effect. In addition, we found that *Olig2* transcripts were not up-regulated in our *Olig1* null mice.

**Conclusions:**

Our findings support the original conclusion that Olig1 plays a minor and non-essential role in oligodendrocyte development and have implications for the interpretation of studies based on *Olig1* deficient mice (and perhaps *Olig1-Cre* mice) from different sources.

## Background

*Oligodendrocyte lineage* genes *Olig1* and *Olig2* encode basic helix-loop-helix (bHLH) transcription factors. Olig2 is a master regulator of oligodendrocyte (OL) lineage development [1-3]. Olig2 is also required for generation of some neurons, notably spinal motor neurons (MNs) [1-3]. MNs are generated from neural stem/progenitor cells in a specialized region of the ventral ventricular zone (VZ) of the spinal cord known as pMN. Around embryonic day 12 (E12) in mice, the same group of progenitors stops producing MNs and switches to production of OL precursors (OPs), which proliferate and migrate away from the VZ in all directions before associating with axons and differentiating into myelin-forming OLs (reviewed in reference [4]). Olig1 and Olig2 (referred to here as Oligs) are involved at multiple stages of this developmental sequence. Olig2 is also required for specifying oligodendrocytes and some types of neurons in the brain – some ventrally-derived interneurons and cholinergic projection neurons in the forebrain, for example [5].

Olig1 can compensate for Olig2 in some regions including the hindbrain and parts of the forebrain, because OPs still form there in *Olig2* null mice but not in *Olig1/Olig2* double nulls [1,3]. Olig1 also plays a later role in the differentiation of OPs into myelinating OLs, although there is disagreement about whether there is an absolute requirement for Olig1 during normal development [1,6]. The original *Olig1* null allele, made by inserting a *Cre-frt-Pgk-Neo-frt* cassette into the mouse *Olig1* locus [1] caused a delay in the appearance of differentiated OLs but no long-term myelin deficit. However, a subsequent study by Xin et al. [6], who crossed the original line with FLP-expressing mice to remove the *Pgk-Neo* selection cassette (leaving behind *Olig1-Cre*), found a severe myelination defect leading to early postnatal lethality. Apart from this contested role in OL lineage development, Olig1 is known to be required for remyelination of experimentally-induced demyelinated lesions in the mouse spinal cord [7].

Given the central role of the Oligs in OL lineage development, it is important to try to settle the controversy over the developmental requirement for Olig1. This might have added significance because the *Olig1* null locus [1,6] contains an expressed Cre cassette under *Olig1* transcriptional control and these *Olig1(+/Cre)* mice are being used to delete floxed genes specifically in OL lineage cells. For example, conditional deletion of *Dicer1 (flox/flox)* using *Olig1(+/Cre)*[6] caused severe impairment of myelination and death around P21 [8], whereas analogous experiments using *Olig2(+/Cre)* or *Cnp(+/Cre)* resulted in only slightly delayed myelination with full recovery by P60 [9]. In another example, constitutively activating the Wnt signaling pathway by conditional deletion of exon 3 of *β-catenin*[10] using *Olig1(+/Cre)* completely prevented OL lineage specification, judging by the complete absence of OP markers such as Pdgfra [11], whereas similar experiments using *Olig2(+/Cre)* did not affect OP specification but only their subsequent differentiation into OLs [12]. While there might be a simple explanation for these differences, such as earlier or more complete recombination by *Olig1(+/Cre)* than by *Olig2(+/Cre)*, the possibility remains that the *Olig1* null allele generated by Xin et al. [6] might carry some additional, unidentified defect that can amplify the phenotype of other deleterious mutations.

To attempt to throw some light on these matters we undertook a study of two independent *Olig1* null lines generated in our own laboratory. We found that loss of Olig1 causes a transient delay in OL development and myelination. We quantified *Olig2* mRNA in our *Olig1* mutant mice and found no increase relative to wild type controls. The mild phenotype we observe is therefore likely to be a genuine consequence of Olig1 loss, not moderated by *cis* regulatory effects on *Olig2*.

## Methods

### Mice

Mice were maintained on a 12 hour light–dark cycle. For timed mating, male and female mice were caged together overnight (from ~6 pm) and 12 noon the following day was designated embryonic day 0.5 (E0.5). All mouse work was approved by the Home Office of the UK Government, and conformed to the Animals (Scientific Procedures) Act 1986. New *Olig1* null lines, *Olig1(-/-)* and *Olig (-/-), Olig2(Tg)* were generated as described previously [13] (also see Results).

### Embryonic Stem (ES) cell targeting

**Figure 1:**
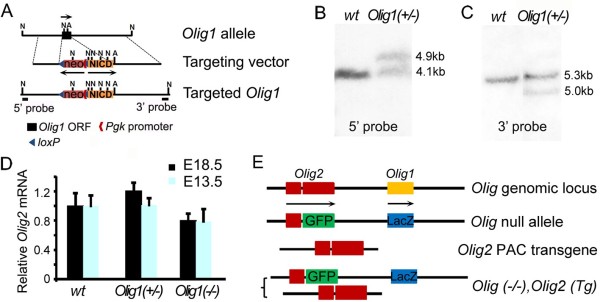
**Generation of *Olig1 *null mouse lines. (A)** The *Olig1* ORF was replaced by Notch1 intracellular domain (NICD) by homologous recombination in ES cells followed by blastocyst injection to generate *Olig1(-/-)* mice. A *Pgk-Neo* cassette, flanked by *loxP* sites, was inserted just upstream of NICD. Arrows indicate the 5’-3’ directions of each coding region. **(B)** Southern blot of NcoI-digested genomic DNA using a 700 bp 5’ probe revealed a 4.1 kb band in wild type mice. An additional 4.9 kb band was identified in heterozygous mice. **(C)** Southern blots of NcoI/AscI-digested genomic DNA using a 200 bp 3’ probe revealed a 5.3 kb band in wild type mice. An additional 5 kb band was identified in heterozygous mice due to the introduction of a new AscI site in the targeting vector as part of the cloning procedure. **(D)***Olig2* expression was not up-regulated in *Olig1(-/-)* mice. Quantitative PCR using cDNA templates acquired from E13.5 and E18.5 forebrain tissues revealed that *Olig1* heterozygotes and homozygous knockout mice expressed similar amounts of *Olig2* mRNA. **(E)** As an alternative approach to generating *Olig1* null mice, *Olig2* PAC transgenic mice (generated by pronuclear injection; [13]) were crossed to *Olig1/2* double KO mice to rescue *Olig2* and produce [*Olig(-/-), Olig2(Tg)*] mice, which phenocopy *Olig1(-/-)*. Arrows show the direction of transcription.

We generated a new *Olig1(-/-)* line by ES cell targeting. Briefly, *Olig1* targeting vector (see Results) was linearized and electroporated into R1 ES cells (129 background) [14]. After 10 days’ selection in 150 μg/ml G418 (Invitrogen), 200 colonies were picked and expanded in 96-well plates. Targeted ES clones were identified by Southern blotting using a 700 bp NcoI—EcoRI fragment as probe (Figure 1B). Positive ES clones were confirmed by Southern blotting using a 200 bp PstI—NcoI probe (Figure 1C). Five correctly targeted ES cell clones were expanded for karyotyping; two clones with normal karyotype were used for C57/B6 blastocyst injection to produce chimeric mice. Male chimeras were bred to C57/B6 females to produce *Olig1* heterozygotes.

### Tissue preparation and histochemistry

Embryonic and postnatal spinal cords or brains were immersion-fixed in 4% (w/v) paraformaldehyde in phosphate-buffered saline (PBS) overnight at 4°C. The tissue was cryo-protected overnight at 4°C in 20% (w/v) sucrose in PBS. Tissue was embedded in OCT compound (Tissue-Tek), rapidly frozen on dry ice/isopentane and stored at -80°C. Tissue was cryo-sectioned (nominal thickness 30 μm) in a Bright cryotome and sections collected on Superfrost Plus slides. Sections were treated with blocking solution [10% (v/v) fetal calf serum in 0.1% (v/v) Triton X-100 in PBS] at 20-25°C for one hour before immuno-labeling. Primary antibodies were anti-Sox10 (guinea pig, 1:4,000 dilution; a gift from M. Wegner, University of Erlangen) and anti-Olig1 (rabbit, 1:4,000 dilution; a gift from Charles Stiles, Dana Farber Cancer Institute, Harvard Medical School). Secondary antibodies were Alexa Fluor 488 conjugated goat anti-rabbit and Cy3-conjugated goat anti-guinea pig IgG (Chemicon, 1:500 dilution). Sections were counterstained with Hoechst 33258 dye (Sigma, 1000-fold dilution), for 10 minutes at 20-25°C after the secondary antibody and mounted under coverslips in fluorescence mounting medium (Dako).

Our fluorescence in situ hybridization procedure has been described before; detailed protocols are available at http://www.ucl.ac.uk/~ucbzwdr/Richardson.htm*.* Briefly, digoxigenin (DIG)-labelled RNA probes were transcribed in vitro from cloned cDNAs of *Mbp* or *Plp*. After hybridization, the DIG signal was detected using horseradish peroxidase (HRP)-conjugated anti-DIG (Roche) followed by developing in fluorescein-tyramide reagent (NENTM Life Science Products, Boston).

### Quantitative PCR

Quantitative PCR (qPCR) was performed using forebrain and spinal cord tissue collected from *Olig1* null mice and control littermates that carried either one or two endogenous copies of *Olig1* at embryonic day 13.5 (E13.5) and/or E18.5. The tissue was homogenized in the presence of Trizol reagent (Invitrogen), and total RNA was purified and used for cDNA synthesis following the manufacturer’s instructions. Oligonucleotides 5′ att gta caa aac ggc cac aa 3′ and 5′ agt gct ctg cgt ctc gtc ta 3′ were used for *Olig2* cDNA amplification. Oligonucleotides 5′ aca act ttg gca ttg tgg aa 3′ and 5′ gat gca ggg atg atg ttc tg 3′ were used to amplify *Gapdh* as an internal control. qPCR values were calculated using the relative standard curve method. At least three embryos of each genotype were analyzed at each age.

### Mouse embryonic fibroblast (MEF) culture and Western blotting

Mouse embryos (E13.5-E15.5) were placed in PBS (without Mg or Ca) and the head, vertebral column, dorsal root ganglia, and inner organs were removed. The remaining tissue was digested in 0.25% (w/v) trypsin, finely minced with a razor blade and incubated at 37°C for 15 minutes to make a single-cell suspension. Cells were then plated in 35 mm dishes coated with 0.1% (w/v) gelatin and grown at 37°C in 5% (v/v) CO_2_ in MEF medium (DMEM-Glutamax, 10% FBS, 1:100 MEM non-essential amino acids and 1:1000 2-mercaptoethanol, Invitrogen). A plasmid encoding Cre under the control of the PGK promoter (*pPGKcreSV40*) was used for transfection with Fugene 6 (Promega). Proteins from transfected MEFs and mouse spinal cord tissue were separated by SDS-PAGE and transferred to polyvinylidene difluoride membranes. Rabbit anti-Myc antibody was purchased from Abcam and used at a 1:10,000 dilution. Protein bands were visualized by chemi-luminescence (ECL reagent; GE Healthcare).

## Results

### Generation of new *Olig1* null mouse lines

To try to resolve the discrepancy between the reported phenotypes of two different *Olig1* null mouse lines [1,6] we generated two new *Olig1* null strains, using different approaches. For one, we replaced the Olig1 open reading frame (ORF) with a DNA fragment including an inverted phosphoglycerate kinase promoter -neomycin resistance cassette (*Pgk-Neo*) flanked by *loxP* sites in mouse embryonic stem (ES) cells, line R1 [14] (Figure 1A). We refer to this line as *Olig1(-/-)*. For purposes unrelated to the work described here, the modified locus also included a Myc-tagged Notch intracellular domain (NICD) coding sequence downstream of the floxed *Pgk-Neo* cassette; in the absence of Cre recombinase this NICD cassette is not expressed (Additional file 1: Figure S1) and is phenotypically neutral. Targeted clones were identified by Southern blot analysis of genomic DNA using a 700 bp NcoI–EcoRI fragment as a 5′ probe (Figure 1B). Correct targeting was confirmed using a 200 bp PstI-NcoI fragment as a 3′ probe (Figure 1C). One karyotypically normal ES cell line was selected for blastocyst injection and germline transmission.

Our second *Olig1* KO was generated by crossing *Olig1/Olig2* double-null mice [3] with a phage artificial chromosome (PAC) transgenic line that contains a single copy of mouse *Olig2*[13] (Figure 1E). We refer to this line as *Olig (-/-), Olig2(Tg)*. We confirmed that this line does not express Olig1 protein (Additional file 2: Figure S2).

### Lack of compensatory up-regulation of Olig2 in *Olig1*-null mice

The *Olig1* and *Olig2* genes are located about 40 kb apart on mouse chromosome 16 and there is a large degree of overlap in their expression patterns in vivo [15]. Xin et al. [6] suggested that the *Pgk-Neo* cassette introduced by Lu et al. [1] to disrupt the *Olig1* ORF might have exerted a cis-activating effect on the neighbouring *Olig2* locus, resulting in over-expression of Olig2 which compensated for loss of Olig1. Since our own *Olig1(-/-)* mice also contain a *Pgk-Neo* cassette at the *Olig1* locus (but in the opposite orientation to the mice described in reference 1), we compared Olig2 mRNA levels in our Olig1(-/-) mice and Olig1(+/-) controls. We collected forebrain tissue at two embryonic stages (E13.5 and E18.5) and quantified *Olig2* transcripts by PCR, using total cellular RNA as substrate. We could not detect a significant difference in the brain or spinal cord between *Olig1(-/-)* and *Olig1(+/-)*, indicating that *Olig2* was not mis-regulated by the *Pgk-Neo* cassette at the *Olig1* locus in our mice (Figure 1D and Additional file 3: Figure S3).

Note that although two *Pgk-Neo* cassettes are present at the disrupted *Olig1/Olig2* locus in our *Olig(-/-), Olig2 (Tg)* mice (Figure 1E) they are almost certainly physically remote from the randomly-integrated *Olig2* PAC transgene and therefore are not expected to impose cis-regulation on *Olig2*.

### Oligodendrocyte precursors are specified normally in *Olig1* null CNS

We analyzed the expression of both platelet-derived growth factor receptor-alpha (Pdgfra), a marker of OPs, and Sox10, which marks all stages of the OL lineage, by immunofluorescence microscopy of E15.5 spinal cord and P2 forebrain sections. Neither Pdgfra nor Sox10 expression were noticeably altered in our two *Olig1* null lines, relative to *Olig1(+/-)* controls (not shown). This is as expected, given that Olig1 protein does not appear until after OP specification [16], and is consistent with the phenotypes of the two previously-described *Olig1* null lines [1,6].

### OL differentiation is delayed in *Olig1* null spinal cord

**Figure 2:**
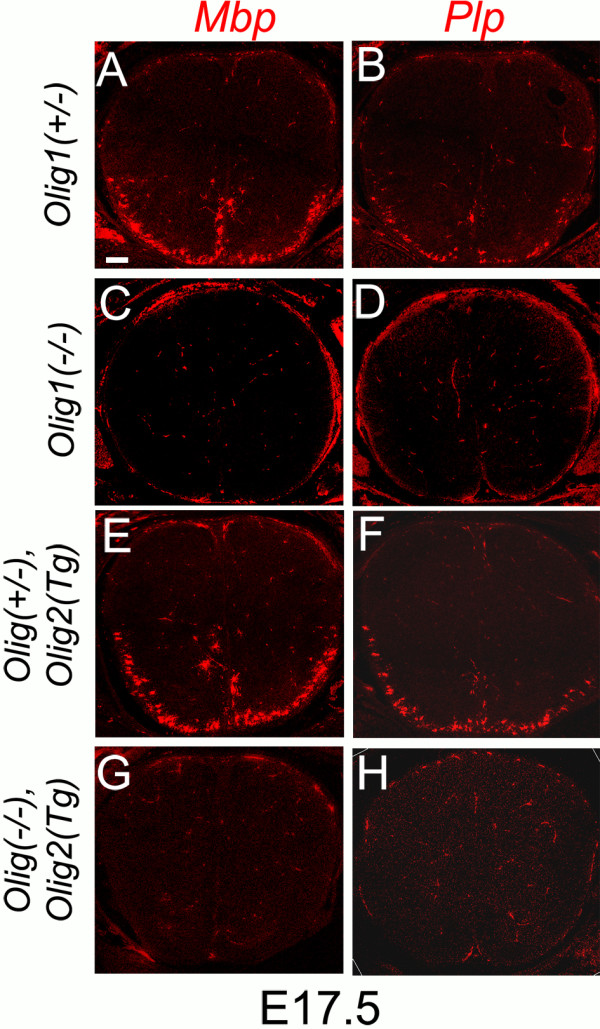
**No OL differentiation in E17.5 *Olig1 *null spinal cord.** Fluorescence in situ hybridization for myelin basic protein (*Mbp*) **(A,C,E,G)** or proteolipid protein (*Plp*) **(B,D,F,H)** gene transcripts was performed on sections of E17.5 mouse spinal cords. No *Mbp-* or *Plp*-positive cells were detected in the circumferential white matter of *Olig1(-/-)* or *Olig(-/-),Olig2(Tg)* cords (**C,D** and **G,H** respectively) compared to cognate *Olig1(+/-)* controls **(A,B and E,F)**. Scale bar: 80 μm.

**Figure 3:**
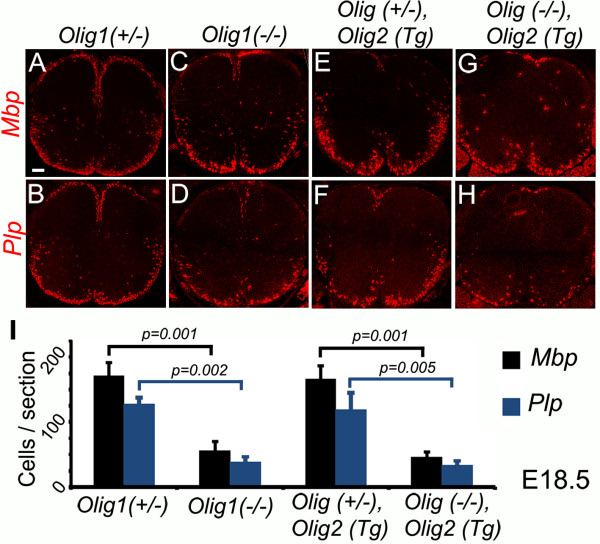
**Reduced OL numbers in E18.5 in *Olig1 *null embryos.** The levels of *Mbp***(C,G)** and *Plp***(D,H)** expression were decreased in *Olig1* null spinal cords compared to corresponding controls (**A,E** and **B,F** respectively). Differentiating OLs were quantified based on *Mbp* and *Plp* expression **(I)**. Three sections per embryo (n = 3) were counted and the data presented as mean ± s.e.m. *p* values (*p* < 0.05) were calculated by Student’s *t*-test. Scale bar: 80 μm.

**Figure 4:**
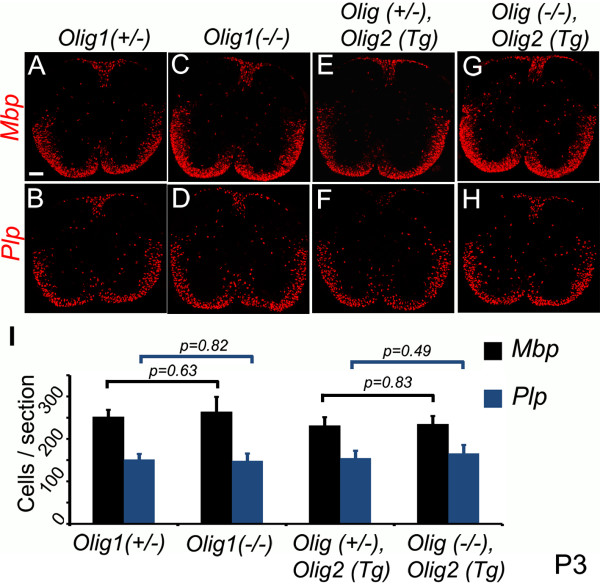
**OL numbers recover by P3.** At P3, the numbers of *Mbp*-and *Plp*-expressing cells in *Olig1* null spinal cords **(C,D and G,H)** were indistinguishable from controls **(A,B** and **E,F** respectively). Differentiating OLs were quantified based on *Mbp* and Plp expression **(I)**. Three sections per mouse (n = 3) were counted and the data displayed as mean ± s.e.m. *p* values (*p* < 0.05) were calculated by Student’s *t*-test. Scale bar: 100 μm.

To investigate OL differentiation in our two new Olig1 null lines, we visualized mRNAs encoding mature OL markers myelin basic protein (MBP) and myelin proteolipid protein (PLP) by in situ hybridization. At E17.5, *Mbp* and *Plp* transcripts were absent from spinal cord in both *Olig1* null lines, in contrast to littermate controls that carried one good copy of endogenous *Olig1* (Figure 2). At E18.5, *Mbp* and *Plp* transcripts were present but in lower numbers of cells relative to Olig1 0heterozygotes (Figure 3); by postnatal day 3 (P3), there were normal numbers of *Mb*p and *Plp*-positive cells in the *Olig1* null spinal cord (Figure 4). These results indicate that Olig1 is involved in, but is not critically important for OL differentiation in the developing spinal cord, consistent with the original study by Lu et al. [1].

OL differentiation in mouse forebrain does not begin until after birth [17]. On forebrain sections, few *Mbp* and/or *Plp* positive cells were detectable by fluorescence in situ hybridization at P4 (not shown). At P7, both *Olig1* null lines appeared to have normal numbers of *Mbp* and *Plp* positive cells in both the corpus callosum and cortex compared to control mice (Additional file 4: Figure S4).

## Discussion

We generated two new *Olig1* null mouse lines by different routes -one by homologous recombination in ES cells followed by blastocyst injection, and the other by transgenic rescue of a previously generated *Olig1/Olig2* double-null line [3] by pronuclear injection of an *Olig2* PAC. Neither of the *Olig1* null lines showed any evidence of prenatal lethality and both lines lived and reproduced normally. There was a transient delay in the production of differentiated OLs in the spinal cords of both our *Olig1* null lines, as originally reported by Lu et al. [1] but in contrast to Xin et al. [6], who reported a severe myelination block that resulted in death around the third postnatal week. Xin et al. [6] put the discrepancy down to the fact that the original *Olig1* null allele retained a *Pgk-Neo* cassette, speculating that the presence of this highly-transcribed element might have caused compensatory up-regulation of the neighbouring *Olig2* gene. Xin et al. [6] removed the *Pgk-Neo* cassette (which was flanked by frt sites) by crossing the original Lu et al. [1] line with FLP-expressing mice. However, they did not quantify *Olig2* expression in either of the *Olig1* mutants.

A cis-acting regulatory effect of *Pgk-Neo* has been implied in previous studies. For example, the initially reported lethal phenotype of a germ line *Surf1* deletion [18] was later attributed to the effect of *Pgk-Neo* on expression of unidentified genes near the *Surf1* locus, after a second *Surf1* knockout line lacking the *Pgk-Neo* cassette was found to be unusually long-lived [19]. Another example is the germ line knockout of the zinc finger transcription factor *Zfp191*, which was initially reported to be embryonic-lethal [20]. Subsequently, an independent line was found to survive after birth, developing a severe dysmyelinating phenotype and dying around P25 [21]. One potential explanation for the difference was that the embryonic-lethal allele contained an expressed Neo selection cassette. We tested the hypothesis that the mild phenotype of our *Olig1(-/-)* mice might have been due to compensatory up-regulation of the adjacent *Olig2* gene by *Pgk-Neo*, but found no evidence for this. Our data are consistent with a previous study by Samanta et al. [22] who found no evidence for up-regulation of Olig2 when they used the *Olig1(+/Cre)* line of Lu et al. [1] (which also contains *Pgk-Neo*) for conditional deletion of bone morphogenetic protein receptor-1a (BMPR1a). Taken together, the data indicate that the presence or absence of *Pgk-Neo* cannot easily explain the dramatically different developmental phenotypes of different *Olig1* null mice.

Different phenotypic outcomes for the same gene deletion can sometimes result from differences in the genetic backgrounds of the mice. For example, the effect of knocking out Nogo-A, a membrane protein of the adult myelin sheath and an inhibitor of neurite growth and axon regeneration, has a much larger effect on neurite regeneration ability in the 129X1/SvJ background than in the C57BL/6 J (C57) background [23]. Our *Olig1(-/-)* line was generated using R1 ES cells (129 background; reference [14]). Homozygous nulls were maintained in a 129/C57 mixed background for many (>10) generations with no sign of lethality. They are now maintained on a 129/C57/CBA background, also with no sign of lethality. The *Olig1* null of Lu et al. [1] was made using J1 ES cells (129) and crossed onto C57 for analysis. The background of our *Olig(-/-),Olig2(Tg)* line is mixed C57/CBA and these mice also display a mild phenotype. The line displaying the contradictory lethal phenotype made by Xin et al. [6] was a modification of Lu et al.’s [1] line, maintained in a mixed 129/C57 background. Altogether, there is no compelling reason to think that genetic background underlies the differing severity of *Olig1* disruption in different lines.

Another possible reason for the divergent phenotypes reported by Lu et al. [1] and Xin et al. [6] might lie in the way in which their mouse lines were generated. Xin et al. [6] made their line by crossing the mice made previously by Lu et al. [1] with a line that expresses FLP recombinase ubiquitously, in order to effect germ line excision of the *frt*-flanked *Pgk-Neo* cassette. Given that *Olig1* and *Olig2* lie close to each other on the chromosome (~40 kb apart) and share significant sequence homologies [24], it is conceivable that an unintended recombination event might have taken place, altering the *Olig* locus in some way that affects Olig2 expression or structure in addition to disrupting *Olig1*.

Arnett et al. [7] previously showed that the *Olig1* null line of Lu et al. [1] inefficiently remyelinates demyelinated lesions produced either by focal injection of lysolecithin or by systemic administration of cuprizone, despite the nearly normal developmental time course of myelination of these mice [7]. This implied that Olig2 and Olig1 have complementary roles in myelin development and repair, respectively. We have no reason to question this conclusion and have not tested the remyelination abilities of our new *Olig1* null mice.

OL differentiation is subject to two-tier transcriptional regulation: 1) epigenetic repression of transcriptional inhibitors and 2) direct transcriptional activation of myelin genes [25]. Transcription factors Olig2 [26], Sox10 [27], MRF [28] and Zfp191 [21] are critical for OL differentiation and/or myelination. Ascl1 and Nkx2.2 also play important roles; germ line knockout of either *Nkx2.2* or *Ascl1* leads to decreased expression of myelin genes in neonatal mice, suggesting that both genes can promote OL maturation [17,29]. In the present study, we have confirmed that *Olig1* deletion delays myelin gene expression. In addition, our previous work has shown that Olig1 can synergize with Sox10 to activate *Mbp* gene transcription [30]. Taken together, we believe that OL development is controlled by indispensible core factors (such as Olig2, Sox10, MRF, Zpf191) in conjunction with other factors (such as Olig1, Ascl1 and Nkx2.2) that are not crucial but serve to adjust the timing of OL differentiation.

## Conclusions

Using two newly-generated *Olig1* null lines we show that loss of Olig1 causes a transient delay in OL development and myelination. Our data confirm the original description of a mild phenotypic effect of Olig1 loss [1], but run counter to the subsequent report of a complete myelination block [6]. We have shown that the mild phenotype is unlikely to result from compensatory up-regulation of *Olig2,* as suggested [6]. We conclude that Olig1 is non-essential for OL development.

## Competing interests

The authors declare that they have no competing interests.

## Authors’ contributions

WDR obtained funding. WDR, HL, NTK designed the experiments and interpreted the results. JPdF, HL and PA carried out the experiments. HL drafted the manuscript and WDR helped revise it. All authors read and approved the final manuscript.

## Authors’ information

William D Richardson and Huiliang Li are joint senior authors.

## Supplementary Material

Additional file 1: Figure S1 No NICD expression in *Olig1(+/-)* or *Olig1(-/-)* mice. Proteins from E18.5 spinal cord were subjected to SDS-PAGE, followed by Western blotting with rabbit anti-Myc antibody. pPGKcreSV40-transfected MEFs derived from *Olig1(+/-)* embryos were used as positive control. The NICD band is indicated by an arrow. Click here for file

Additional file 2: Figure S2 Our new *Olig1* null mice do not express Olig1 protein. Co-immunolabeling for Olig1 (green) and Sox10 (red) was performed on sections of E18.5 mouse spinal cords. No Olig1-positive cells were detected in either *Olig1(-/-)* (B, B’) or *Olig(-/-),Olig2(Tg)* spinal cords (D, D′) in contrast to in *Olig1(+/-)* (A, A’) or *Olig(+/-),Olig2(Tg)* controls (C, C′). Scale bar: 80 μm for A-D and 20 μm for A’-D’. Click here for file

Additional file 3: Figure S3 No up-regulation of *Olig2* expression in *Olig1(-/-)* spinal cord. Quantitative PCR using cDNA templates prepared from E18.5 spinal cord tissue revealed that there was no appreciable difference in the expression of *Olig2* mRNA between *Olig1(+/+)* and *Olig1(-/-)* lines or between *Olig(+/-),Olig2(Tg)* and *Olig(-/-),Olig2(Tg)* lines. Click here for file

Additional file 4: Figure S4 No change in OL numbers in *Olig1* null forebrain at P7. In the developing forebrain, OL differentiation starts in the first postnatal week. At P7, coronal sections showed that the numbers of *Mbp*- and *Plp*-expressing cells in *Olig1* null forebrain (B,F and D,H respectively) were similar to those in controls (A,E and C,G respectively). cc, corpus callosum; ctx, cortex. Scale bar: 80 μm. Click here for file
