# Supplementary figures and images for: New Olig1 null mice confirm a non-essential role for Olig1 in oligodendrocyte development
Source: BMC Neurosci. 2014 Jan 14;15:12. doi: 10.1186/1471-2202-15-12 (PMC3904929; doi:10.1186/1471-2202-15-12)

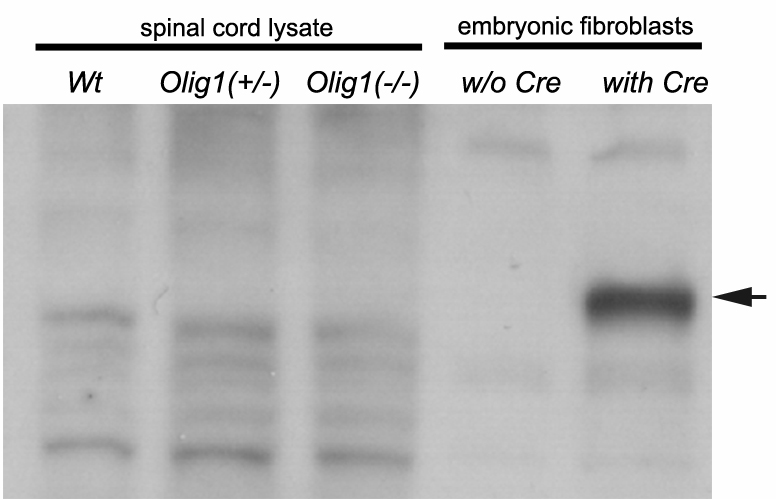

Supplement: Additional file 1: Figure S1 — No NICD expression in Olig1(+/-) or Olig1(-/-) mice. Proteins from E18.5 spinal cord were subjected to SDS-PAGE, followed by Western blotting with rabbit anti-Myc antibody. pPGKcreSV40-transfected MEFs derived from Olig1(+/-) embryos were used as positive control. The NICD band is indicated by an arrow. [file 1471-2202-15-12-S1.jpeg]

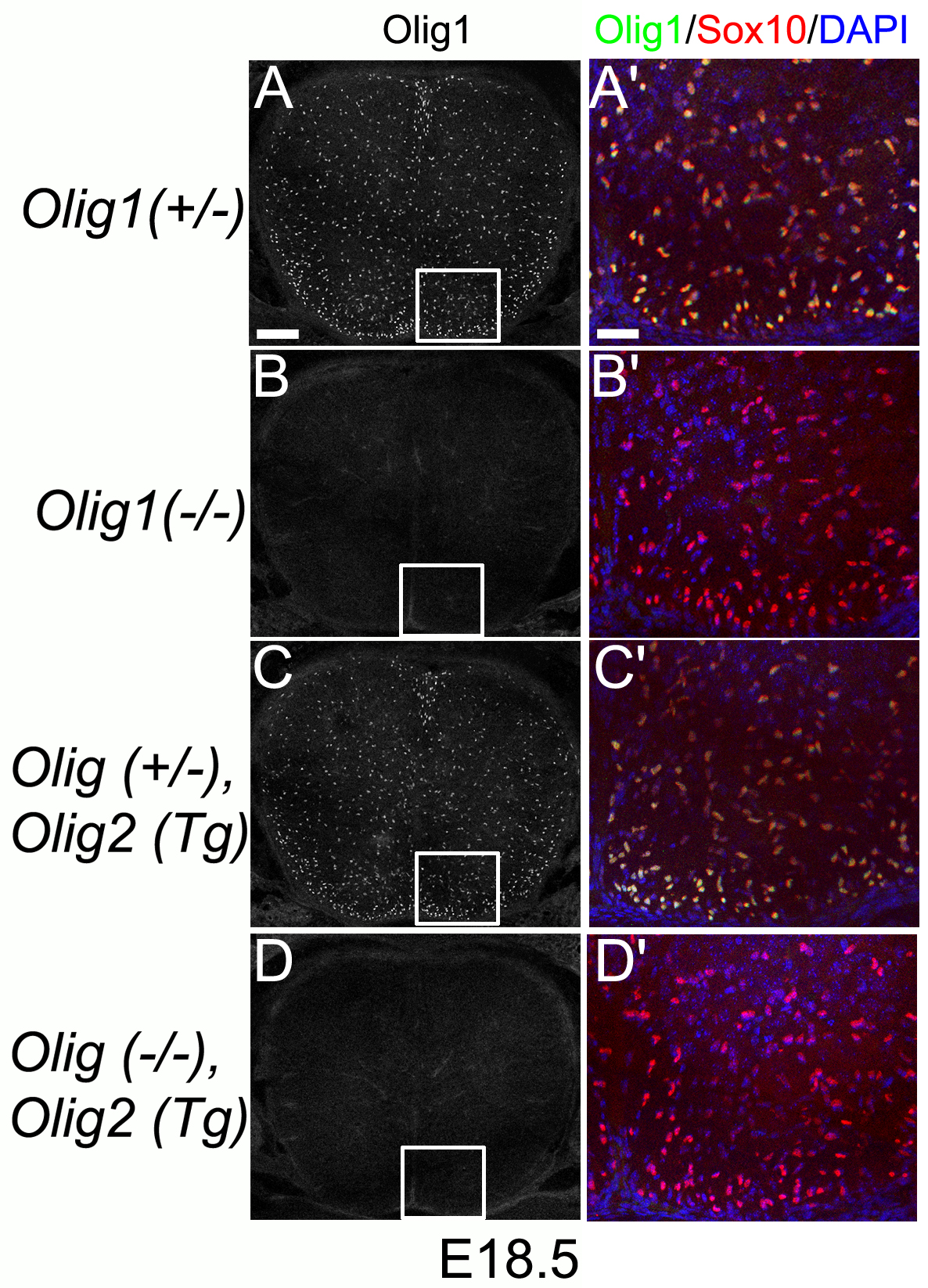

Supplement: Additional file 2: Figure S2 — Our new Olig1 null mice do not express Olig1 protein. Co-immunolabeling for Olig1 (green) and Sox10 (red) was performed on sections of E18.5 mouse spinal cords. No Olig1-positive cells were detected in either Olig1(-/-) (B, B’) or Olig(-/-),Olig2(Tg) spinal cords (D, D′) in contrast to in Olig1(+/-) (A, A’) or Olig(+/-),Olig2(Tg) controls (C, C′). Scale bar: 80 μm for A-D and 20 μm for A’-D’. [file 1471-2202-15-12-S2.jpeg]

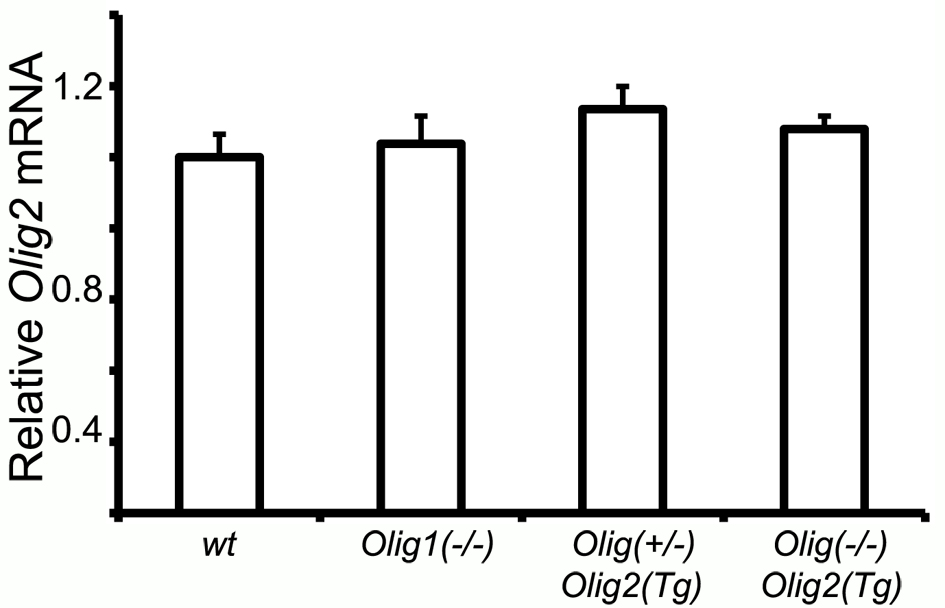

Supplement: Additional file 3: Figure S3 — No up-regulation of Olig2 expression in Olig1(-/-) spinal cord. Quantitative PCR using cDNA templates prepared from E18.5 spinal cord tissue revealed that there was no appreciable difference in the expression of Olig2 mRNA between Olig1(+/+) and Olig1(-/-) lines or between Olig(+/-),Olig2(Tg) and Olig(-/-),Olig2(Tg) lines. [file 1471-2202-15-12-S3.jpeg]

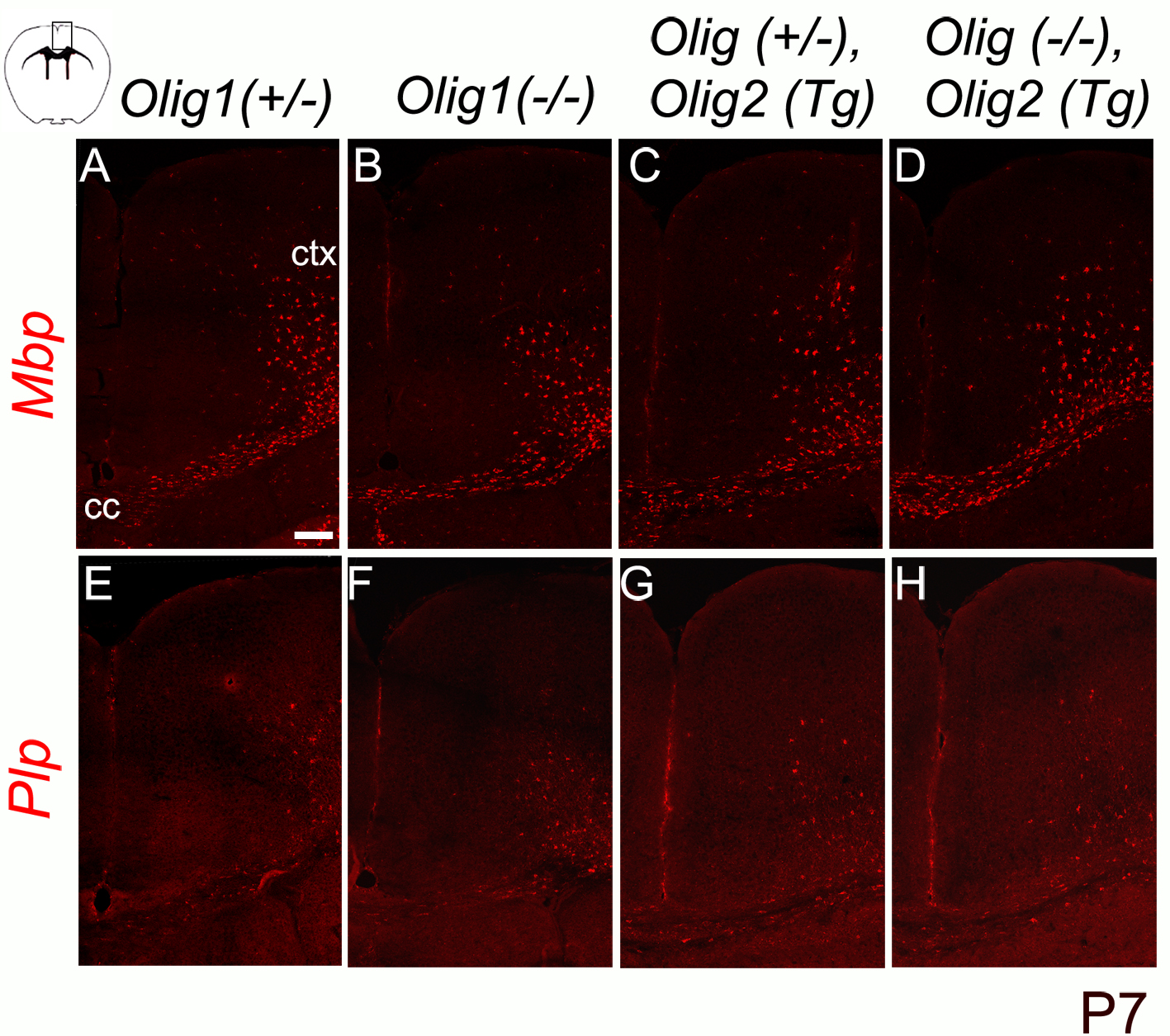

Supplement: Additional file 4: Figure S4 — No change in OL numbers in Olig1 null forebrain at P7. In the developing forebrain, OL differentiation starts in the first postnatal week. At P7, coronal sections showed that the numbers of Mbp- and Plp-expressing cells in Olig1 null forebrain (B,F and D,H respectively) were similar to those in controls (A,E and C,G respectively). cc, corpus callosum; ctx, cortex. Scale bar: 80 μm. [file 1471-2202-15-12-S4.jpeg]
